# Supplementary figures and images for: Screening of faba bean (Vicia faba L.) accessions to acidity and aluminium stresses
Source: PeerJ. 2017 Feb 8;5:e2963. doi: 10.7717/peerj.2963 (PMC5301972; doi:10.7717/peerj.2963)

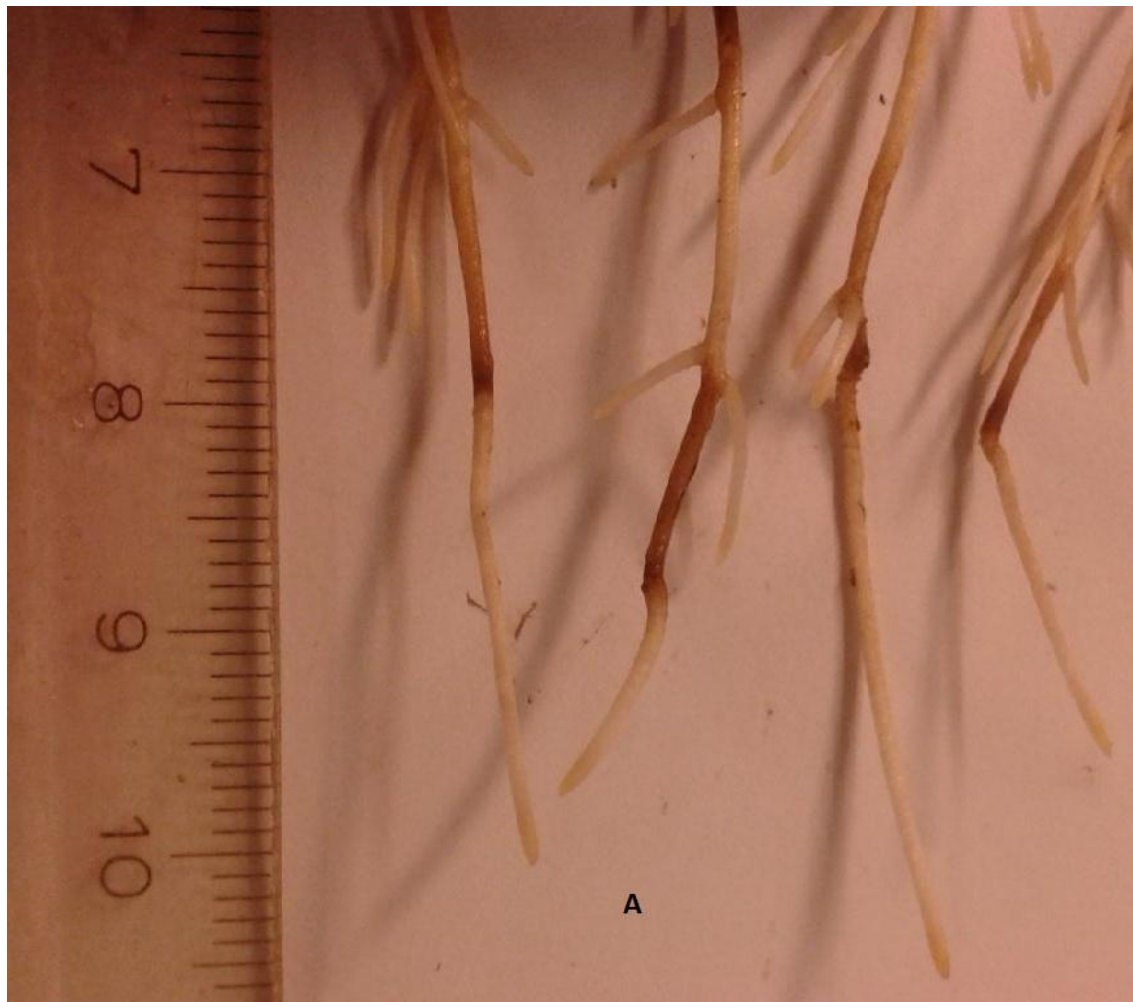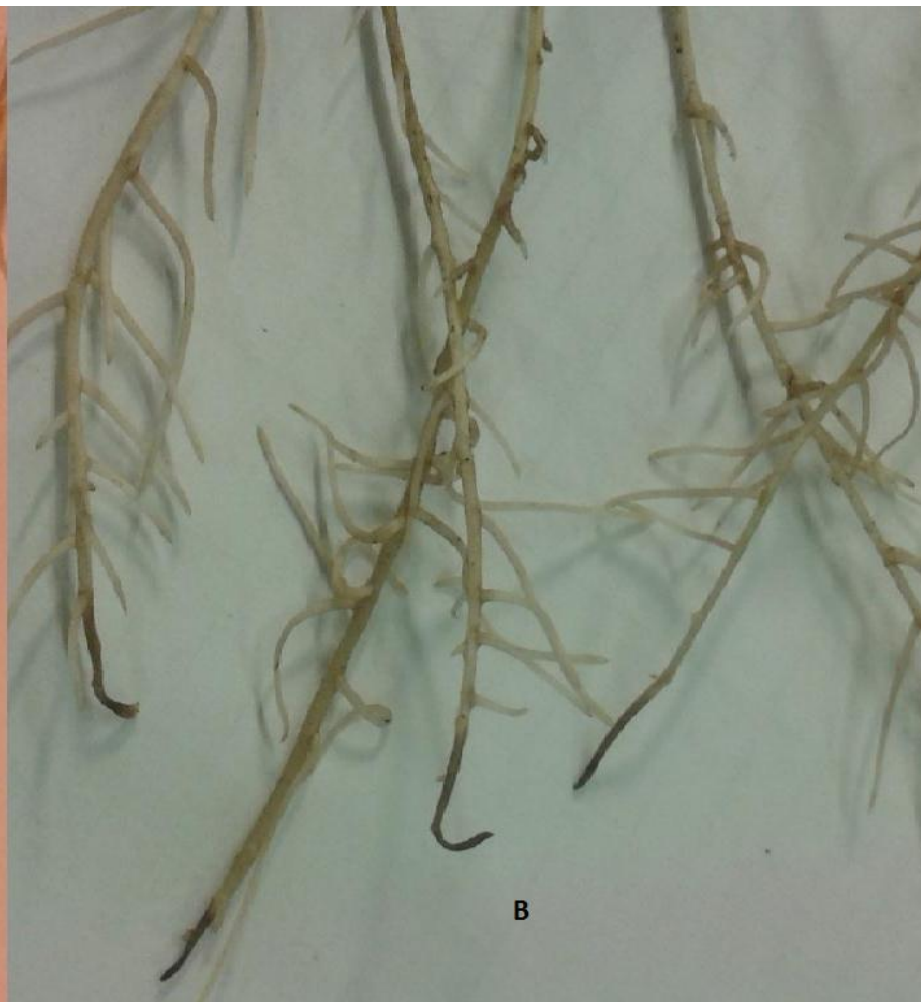

Supplement: Figure S1 — Root regrowth of faba bean seedlings 3 days after transfer from 82 µmol/Laluminium treatment to pH 4.5 Al-free solution. A, accession Hachalu, showing healthy white root tissue beyond the zone of browning caused by the aluminium stress; B, accession Babylon, showing dead brown root tips behind which new laterals formed during recovery. [file peerj-05-2963-s006.pdf]
